# Supplementary material for: The Arabidopsis H3K27me3 demethylase JUMONJI 13 is a temperature and photoperiod dependent flowering repressor
Source: Nat Commun. 2019 Mar 21;10:1303. doi: 10.1038/s41467-019-09310-x (PMC6428840; doi:10.1038/s41467-019-09310-x)
Supplement: Supplementary file 1 — Supplementary Information [file 41467_2019_9310_MOESM1_ESM.pdf]

Supplementary Figures

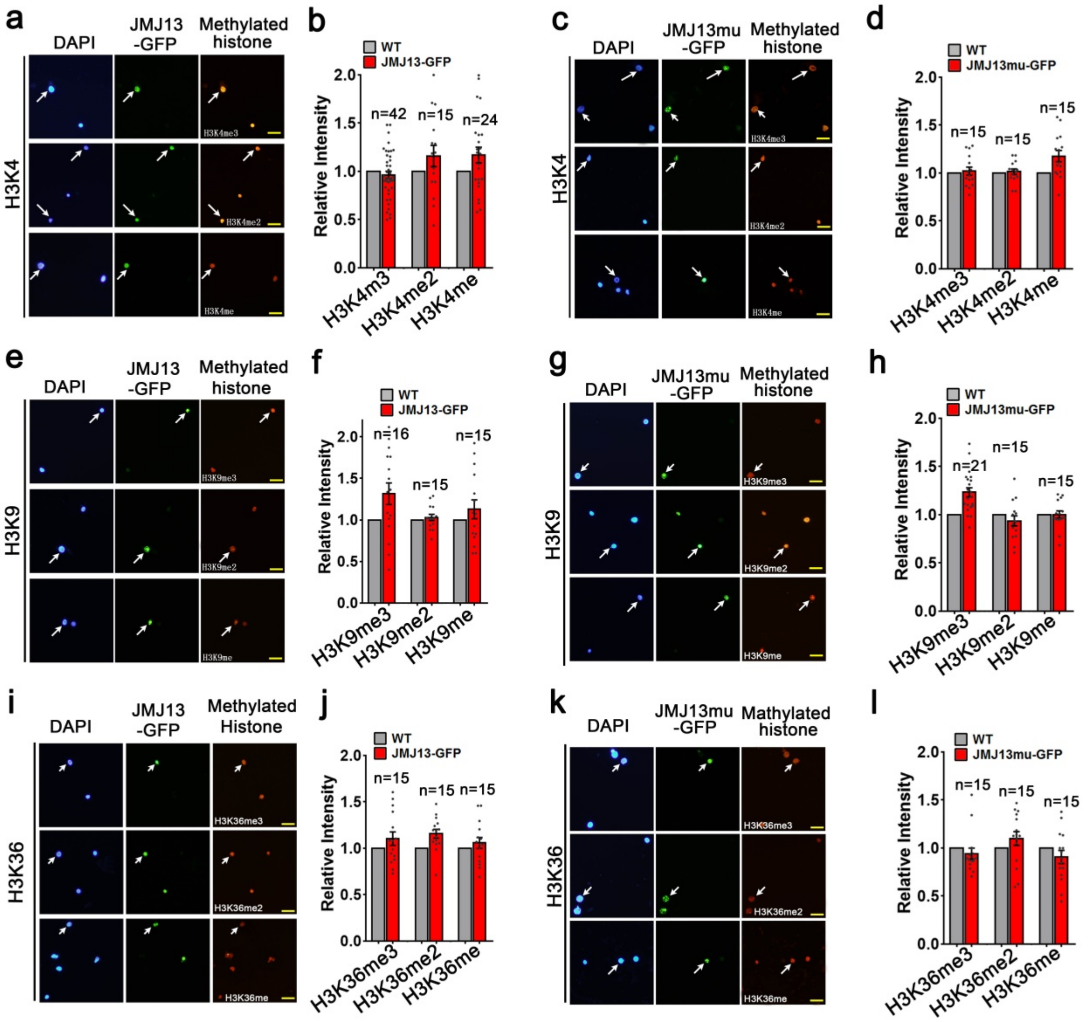

Supplementary Fig. 1. Overexpression of JMJ13-GFP has no effect on H3K4, H3K9, or H3K36 methylation.

a-l H3K4 (**a**, **c**), H3K9 (**e**, **g**), and H3K36 (**i**, **k**) methylation activity was determined by immunostaining with histone modification-specific antibodies. The white arrows point to the transfected nuclei stained by methylation-specific histone antibodies (red, right panels), DAPI (blue, left panels), and the GFP signal from the JMJ13-GFP or JMJ13-H293A-E295A-GFP (green, middle panels), respectively. Scale bars are 2 μm. In **b**, **d**, **f**, **h**, **j,** and **l**, more than 15 pairs of transfected nuclei versus non-transfected nuclei in the same field of view were counted. Error bars indicate mean ± SE. The data points are shown as dots. Source data are provided as a Source Data file.

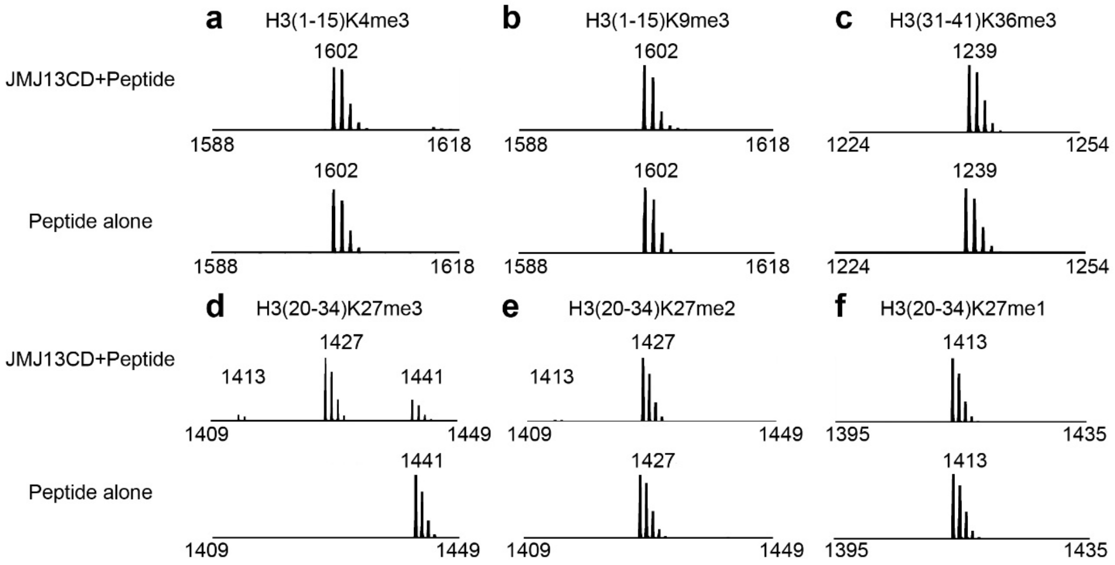

Supplementary Fig. 2. The *in vitro* demethylation assay.

a–f The MALDI-TOF mass-spectrometry based *in vitro* histone demethylation assay of JMJ13CD against histone peptides H3(1-15)K4me3 **(a)**, H3(1-15)K9me3 **(b)**, H3(31-41)K36me3 **(c)**, H3(20-34)K27me3 **(d)**, H3(20-34)K27me2 **(e)** and H3(20-34)K27me1 **(f)** with the JMJ13CD plus the peptide in the upper panel and the peptide alone in the lower panel. The molecular weights of the peaks are labeled. The results show that JMJ13 is predominantly an H3K27me3 demethylase with trace H3K27me2 demethylase activity and no H3K27me1 demethylase activity.

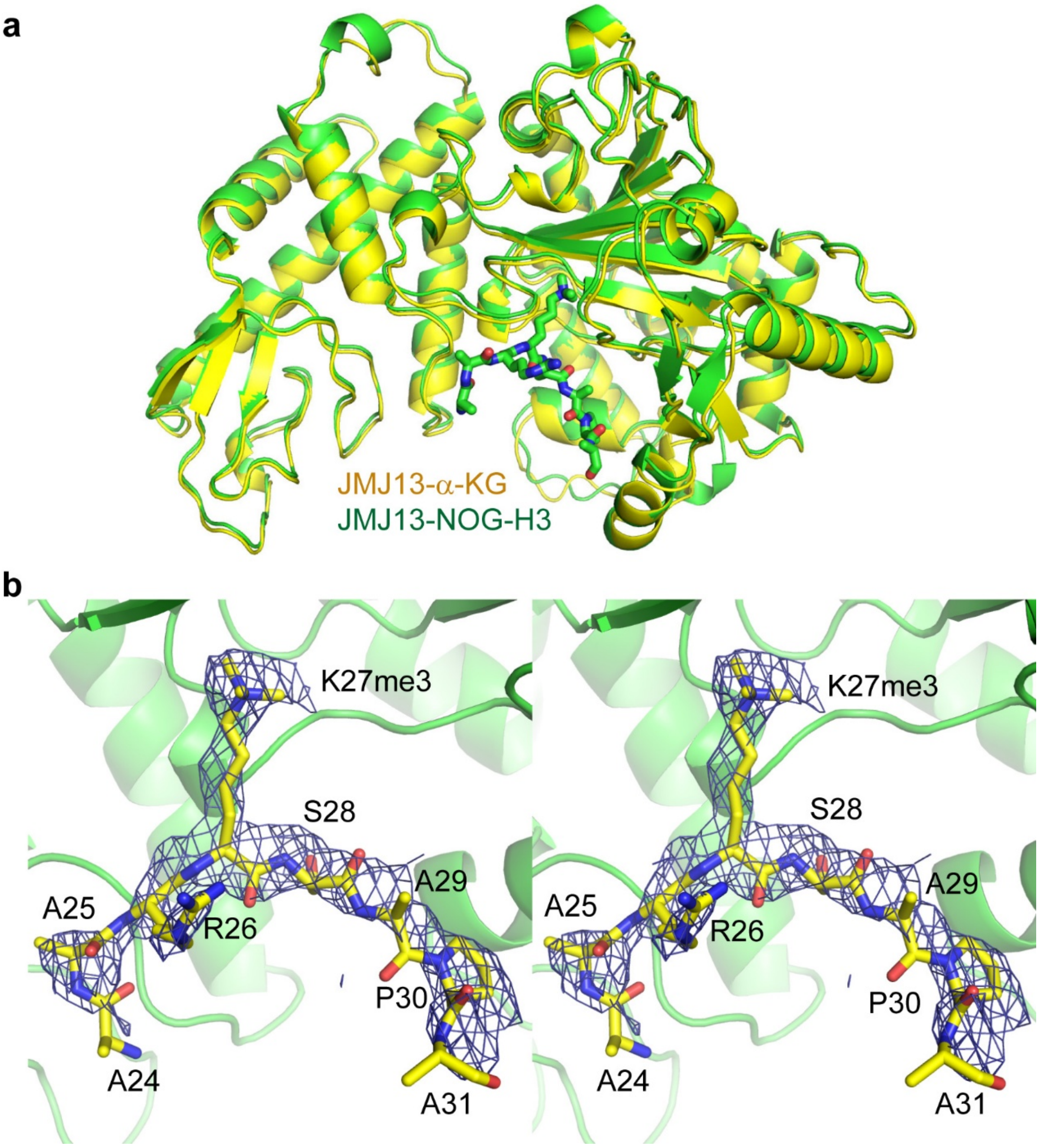

Supplementary Fig. 3. The structure of JMJ13CD.

a Superposition of the JMJ13CD-NOG-H3K27me3 complex (in green) and JMJ13CD-α-KG complex (in yellow) showing no significant overall conformational change. **b** A stereo-view of the SIGMMAA-weighted 2Fo-Fc density map of the H3K27me3 peptide at 0.5 σ level.

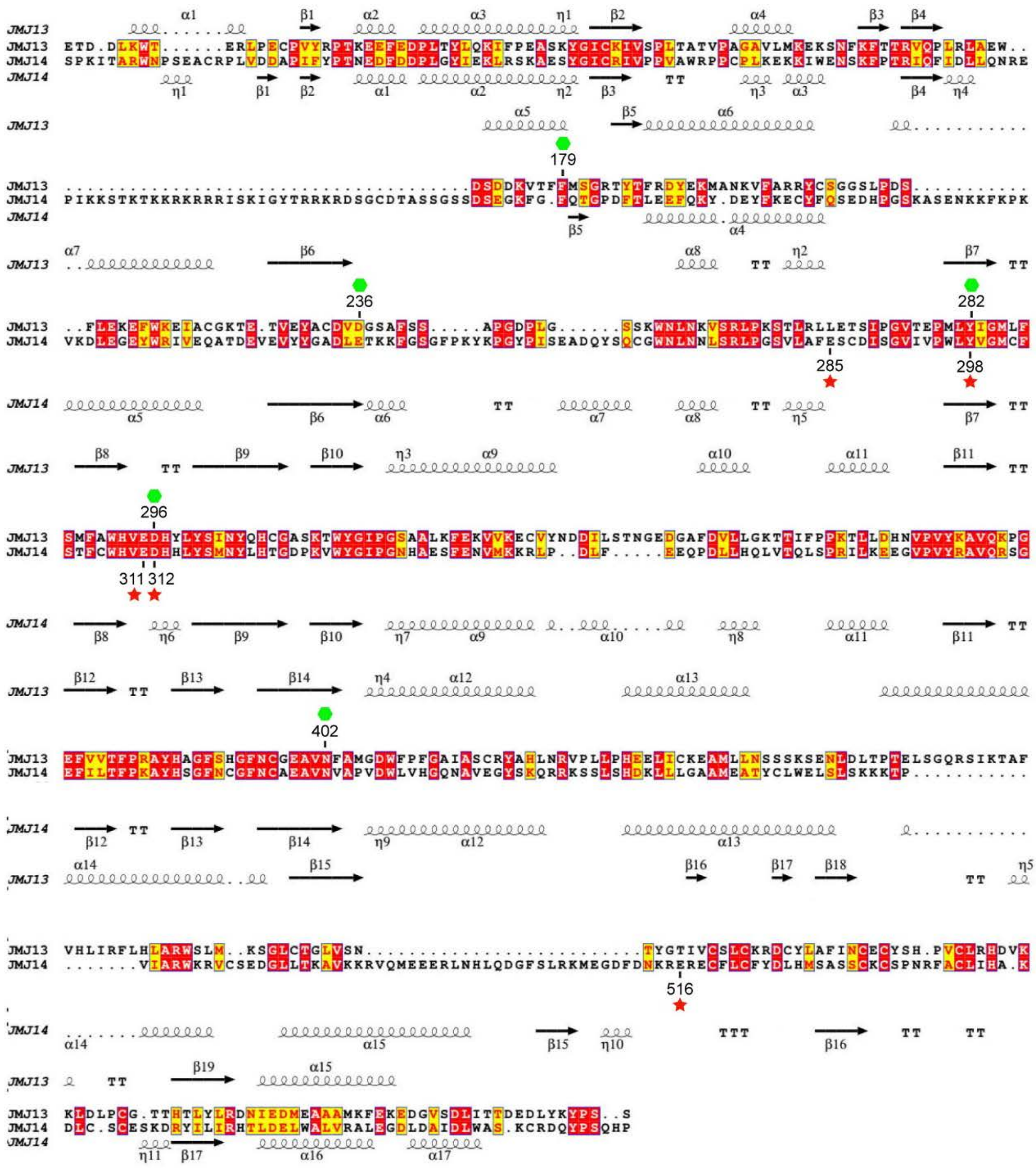

Supplementary Fig. 4. The structure-based sequence alignment of JMJ13CD and JMJ14CD.

The secondary structures of JMJ13CD and JMJ14CD are shown on the top and the bottom of the alignment, respectively. The key residues involved in substrate recognition are highlighted with green hexagons for JMJ13 and red stars for JMJ14.

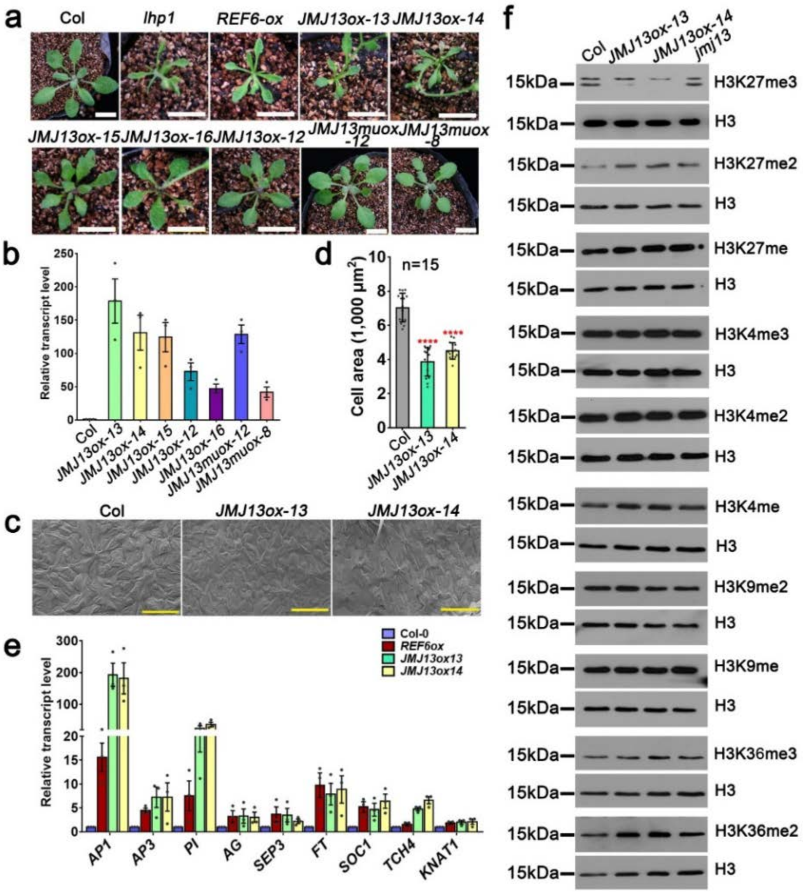

Supplementary Fig. 5. *JMJ13*ox plants display similar phenotypes with H3K27me3 silencing-deficient *lhp1* mutants and *REF6* overexpression plants.

a Phenotype of Col, *lhp1*, *REF6ox*, and *JMJ13ox* plants grown under 22 °C long-day conditions. The five *JMJ13* overexpressing lines showed representative upward curling leaves and various degrees of pleiotropic phenotypes. Weak to strong phenotypes are shown from right to left. The plants carrying the *JMJ13*mu-ox transgene, with mutations in the conserved residues in the enzyme active center did not show the representative phenotypes. White scale bars, 1 cm. The plant images were created by the authors in this research. **b** The relative *JMJ13* expression level was determined by real-time-PCR. Expression levels were normalized to *UBC* (At5g25760). Error bars indicate mean ± SE from three independent experiments. **c** Scanning electronic microscopy shows leaf epidermal cells of Col, and two *JMJ13ox* plants, *JMJ13ox*-13, *JMJox*-14. Yellow scale bars, 100 μm. **d** Statistics of cell area of the epidermal cells. Values are means ± SE of 15 cells. Student's t-test was used to calculate the P value. ****, P < 0.0001. **e** Expression of H3K27me3 target genes in *JMJ13ox* and *REF6ox* plants determined by real-time-PCR. Expression levels were normalized to *UBC*. Error bars indicate mean ± SE from three independent experiments. **f** The global H3 lysine methylation status was changed in *JMJ13ox* plants. Total protein from Col, two *JMJ13*ox plants, and the *jmj13* mutant was examined by immunoblot with the antibodies specified on right. Molecular weight was labeled on left. Immunoblotting with H3 antibody was used as a loading control. The data points are shown as dots. Source data are provided as a Source Data file.

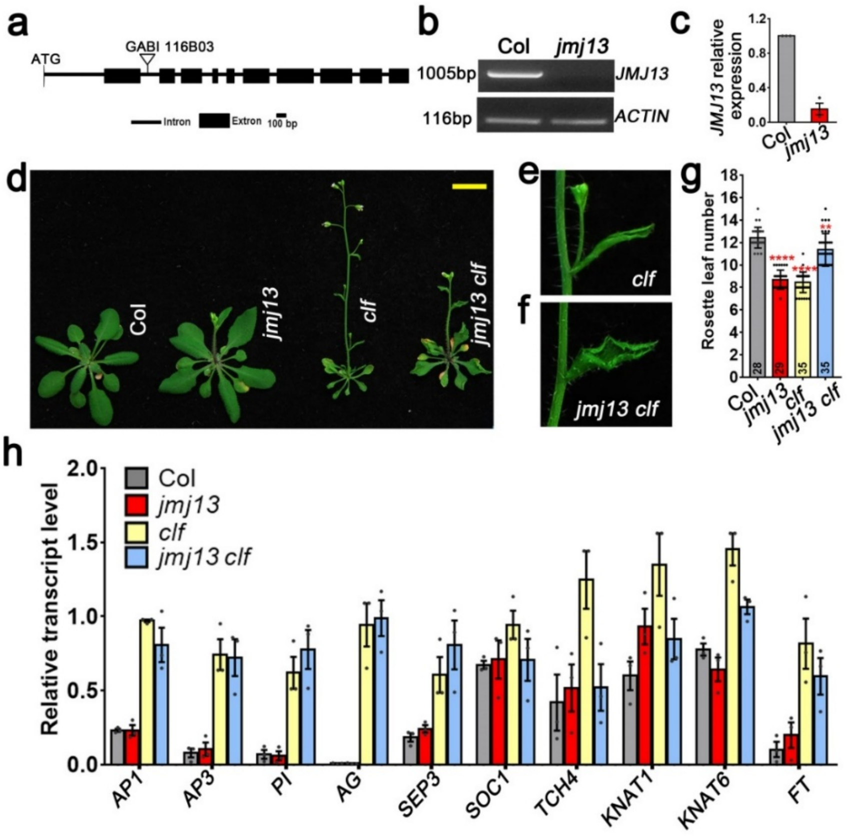

Supplementary Fig. 6. *jmj13* partially suppresses *clf* phenotypes.

a Domain architecture of the *JMJ13* gene. The T-DNA insertion is located in the second intron of *JMJ13*. **b** Semi-quantitative RT PCR to examine the *JMJ13* transcript levels in wild type (Col) and *jmj13* mutant. *ACTIN* was analyzed as the control. **c** Real-time PCR analysis of transcript levels of *JMJ13*. *UBC* was used as the internal reference. Error bars indicate mean ± SE from three independent experiments. **d** Phenotypes of Col, *jmj13*, *clf*, and *jmj13 clf* plants. All the plants were grown under 22 °C LD conditions. Yellow scale bars, 1 cm. **e** and **f** The first cauline leaf of *clf* (**e**) and *jmj13 clf* (**f**). **g** Flowering time was assessed by counting rosette leaves in bolting plants grown under 22°C long-day conditions. Error bars represent mean ± SE of indicated number of plants for three independent biological repeats. Student's t-test was used to calculate the P value. ****, P < 0.0001; **, P< 0.01. **h** Expression of H3K27me3 target genes and FT in Col, *jmj13, jmj13 clf* determined by real-time PCR. Ten-day-old seedlings grown under 22°C LD conditions were collected for total RNA extraction. Expression levels were normalized to *UBC*. Error bars indicate mean ± SE from three independent experiments. The plant images in **d**, **e** and **f** were created by the authors in this research.

The data points are shown as dots. Source data are provided as a Source Data file.

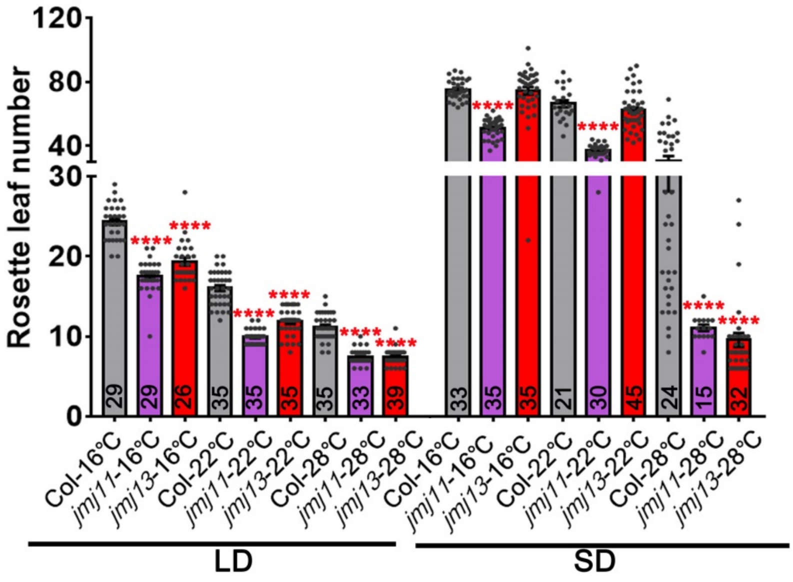

Supplementary Fig. 7. *jmj13* displayed photoperiod- and temperature-regulated flowering phenotypes.

Assessment of flowering time by counting rosette leaves in *jmj11*, *jmj13*, and Col bolting seedlings grown under different temperatures (16°C, 22°C, and 28 °C) and photoperiod conditions (LD and SD). Error bars represent mean ± SE of indicated number of plants for three independent biological repeats. N number was marked in the bottom of the column. Student's t-test was used to calculate the P value. ****, P < 0.0001. The data points are shown as dots. Source data are provided as a Source Data file.

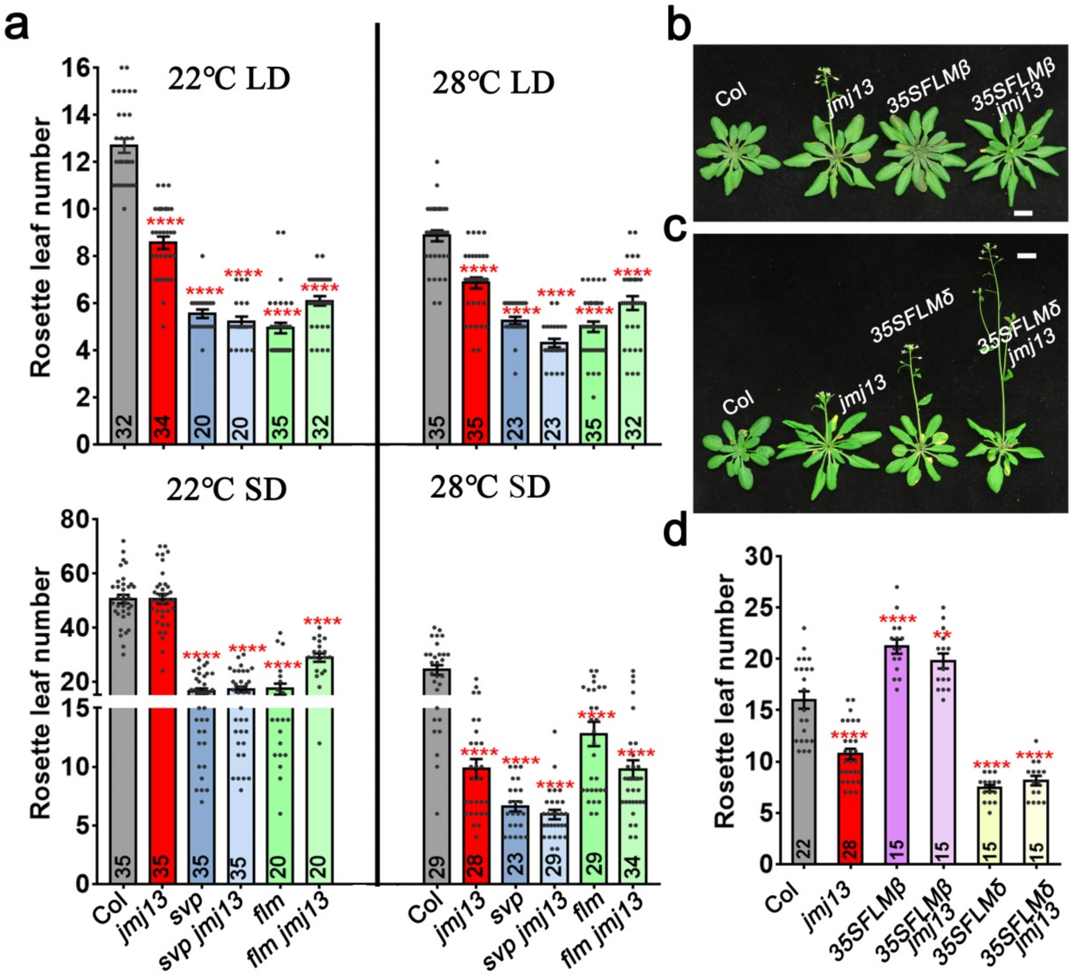

Supplementary Fig. 8. JMJ13 repression of temperature-regulated flowering requires SVP-FLM.

a Assessment of flowering time for Col, *jmj13*, *svp*, *svp jmj13*, *flm* and *flm jmj13* by counting rosette leaf numbers in bolting seedlings grown under different temperatures (22 °C and 28 °C) and different photoperiod (LD and SD). **b** 35S-*FLM*β suppresses early flowering of *jmj13* under 22 °C LD conditions. **c** 35S-*FLM*δ does not suppress early flowering of *jmj13* under 22 °C LD conditions. **d** Rosette leaf numbers in Col, *jmj13*, 35S-*FLM*β (#11), 35S-*FLM*β (#11) *jmj13*, 35S-*FLM*δ (#4), 35S-*FLM*δ (#4) *jmj13* bolting seedlings grown in 22 °C LD conditions. The plant images in **b** and **c** were created by the authors in this research.

Error bars represent mean ± SE of indicated number of plants for three independent biological repeats. N number was marked in the bottom of the column. Student's t-test was used to calculate the P value between wild-type Col and tested lines. ****, $P < 0.0001$; **, $P < 0.01$; scale bars, 1 cm. The data points are shown as dots. Source data are provided as a Source Data file.

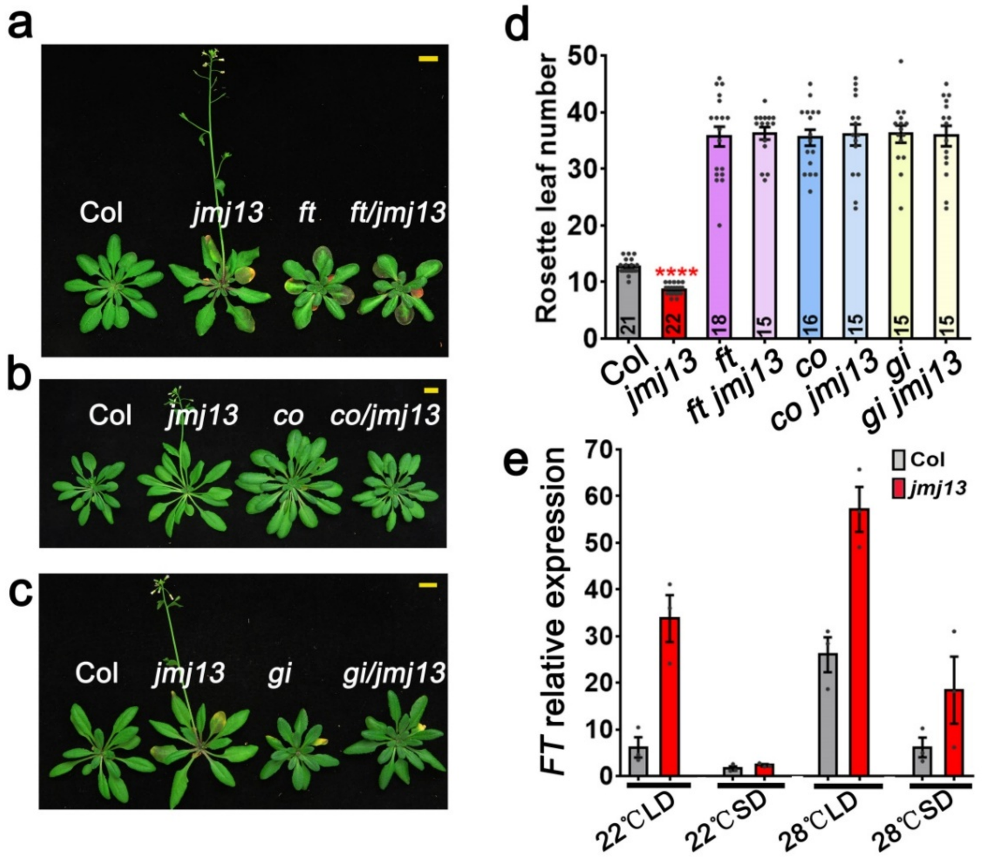

Supplementary Fig. 9. The photoperiod-regulated flowering time by JMJ13 requires CO and GI.

a-c *ft*, *co* and *gi* repressed the early flowering of *jmj13* in 22 °C LD conditions. Scale bars, 1cm. The plant images were created by the authors in this research. **d** The flowering time of Col, *jmj13*, *ft*-1 (CS56), *co*-2 (CS55), *gi*-3 (CS51) and double mutants *ft jmj13*, *co jmj13* and *gi jmj13* were assessed by counting rosette leaf numbers in bolting seedlings grown under 22 °C LD conditions. Values are means ± SE of indicated number of plants for three independent biological repeats. N number was marked in the bottom of the column. Student's t-test was used to calculate the P value between wild-type Col and tested lines. ****, P < 0.0001. **e** The expression of *FT* was analyzed by realtime PCR. Expression levels were normalized to *UBC* (At5g25760). Error bars represent mean ± SE from three independent experiments. The data points are shown as dots. Source data are provided as a Source Data file.

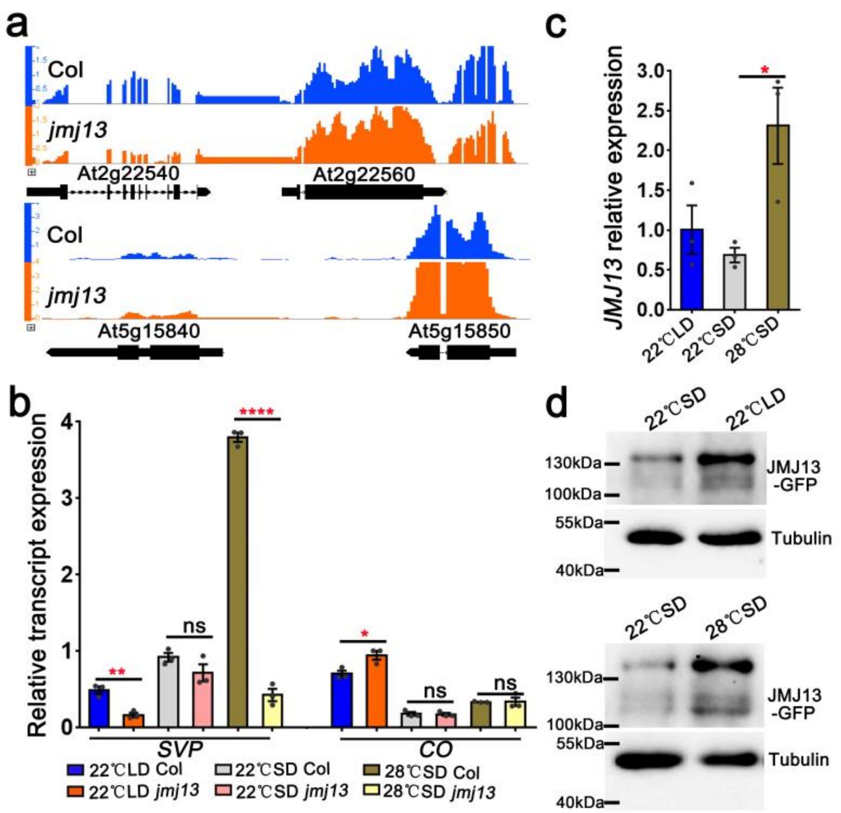

Supplementary Fig. 10. The role of JMJ13 in photoperiod- and temperature-dependent flowering time regulation.

a RNA-seq analysis of the expression of *SVP* (At2g22540) and *CO* (At5g15840) on genome browser. Two neighbor genes (At2g22560 and At5g15850) were shown as control. **b-d** 7-day-old seedlings grown on 22°C SD, 22°C LD and 28°C SD conditions were collected. **b** The transcription level of *SVP* and *CO* was detected by Real-time PCR in wild-type (Col) and *jmj13*. Expression levels were normalized to *UBC* (At5g25760). **c** The transcription level of *JMJ13* was detected by Real-time PCR in wild-type (Col). Expression levels were normalized to *UBC* (At5g25760). **d** The JMJ13 protein accumulation was analyzed by western blotting using the *JMJ13ox-13* plants. The molecular weight marker are labeled on left.

In **b** and **c**, error bars represent mean ± SE from three independent experiments. Student's t-test was used to calculate the P value. ****, $P < 0.0001$; **, $P < 0.01$; *, $P < 0.05$; ns, not significant. The data points are shown as dots. Source data are provided as a Source Data file.

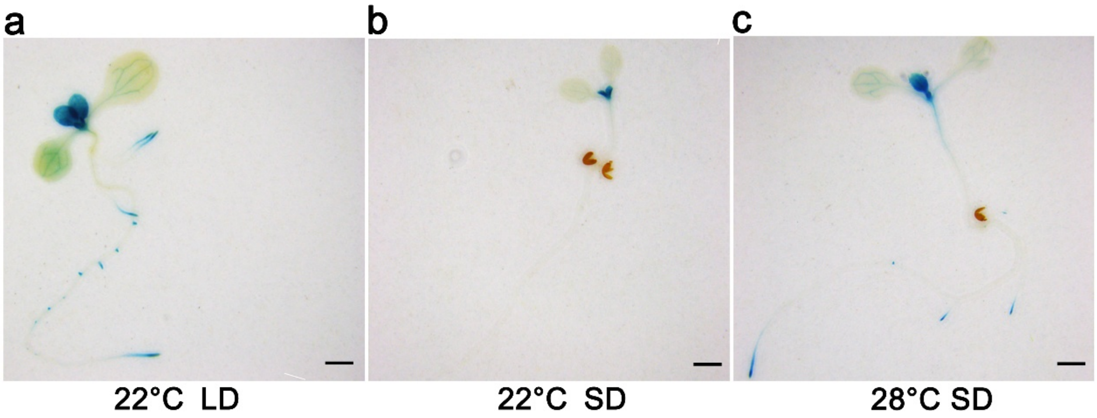

Supplementary Fig. 11. Light and temperature affect *JMJ13* expression.
a–c, Analysis of the expression profile of *JMJ13* using *pJMJ13:JMJ13-GUS* transgenic Col seedlings (blue). Staining was observed using the same homozygous transgenic line in whole 7-day-old seedlings grown under 22°C LD (**a**), 22°C SD (**b**), and 28°C SD (**c**) conditions. Scale bar, 1 mm.

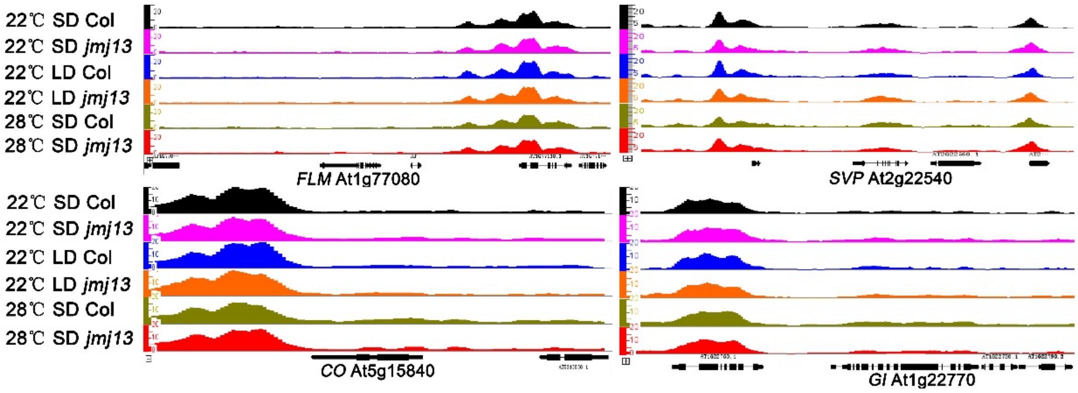

Supplementary Fig. 12. Genome browser view of H3K27me3 signal for different genotypes at the *FLM*, *SVP*, *CO* and *GI* loci. Gene models from TAIR10 are shown at the bottom.

Supplementary Table 1. Comparison of human and Arabidopsis histone demethylases in subfamilies

Subfamily	Species	Enzyme	Substrate	Domain Architecture
KDM4	Human	KDM4A/JHDM3A	H3K9me3, H3K36me3	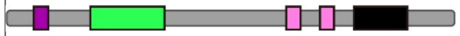
		KDM4B/JHDM3B	H3K9me3	
		KDM4C/JHDM3C	H3K9me3, H3K36me3	
		KDM4D/JHDM3D	H3K9me3, H3K9me2	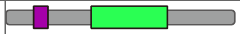
	Arabidopsis	JMJ13	H3K27me3, H3K27me2	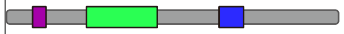
		REF6/JMJ12	H3K27me3, H3K27me2	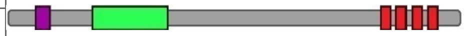
		ELF6/JMJ11	H3K27me3, H3K27me2	
KDM5	Human	KDM5A/JARID1A	H3K4me3, H3K4me2	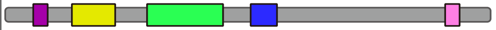
		KDM5B/JARID1B	H3K4me3, H3K4me2, H3K4me1	
		KDM5C/JARID1C	H3K4me3, H3K4me2	
		KDM5D/JARID1D	H3K4me3, H3K4me2	
	Arabidopsis	JMJ14	H3K4me3, H3K4me2	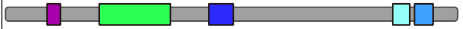
		MEE27/JMJ15	H3K4me3	
		JMJ16	Not determined	
		JMJ18	H3K4me3	
		JMJ17	H3K9me2, H3K9me1	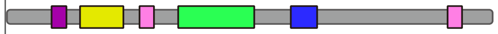
		JMJ19	Not determined	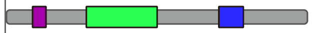
KDM6	Human	KDM6A/UTX	H3K27me3, H3K27me2	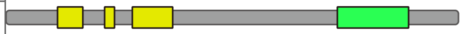
		KDM6C/UTY	H3K27me3, H3K27me2	
		KDM6B/JMJD3	H3K27me3, H3K27me2	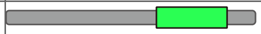
	Arabidopsis	NA	-	-

JmjC JmjN Zf-C5HC2 or Zf-C4HCHC PHD ARID FYRN FYRC Tudor Zf-C2H2

Supplementary Table 2. Data collection and refinement statistics

	JMJ13CD-αKG	JMJ13CD-NOG-H3K27me3
**Data collection**		
Beamline	SSRF-BL19U1	SSRF-BL19U1
PDB code	6IP0	6IP4
Space group	$P6_222$	$P6_222$
Wavelength (Å)	1.2824	0.9793
Cell dimensions		
a, *b*, *c* (Å)	129.7,129.7,231.5	128.8, 128.8, 230.6
α, β, γ (°)	90, 90, 120	90, 90, 120
Resolution (Å)	50.0-2.4 (2.49-2.40) [a]	50.0-2.6 (2.69-2.60)
R_{merge}	0.078 (0.651)	0.083 (0.868)
$I / \sigma I$	36.2 (4.0)	20.9 (1.4)
Completeness (%)	100.0 (100.0)	93.2 (95.1)
Redundancy	19.3 (19.4)	6.8 (4.9)
**Refinement**		
R_{work} / R_{free}	0.192/0.212	0.213/0.247
No. reflections	45,790	33,189
No. atoms	3,963	3,892
Protein/Peptide	3,739/-	3,731/56
Ni^{2+} / Zn^{2+}	1/2	1/2
αKG or NOG	10	10
Water/SO_4^{2-}	146/65	57/35
B-factors (Å^2)	62.9	91.8
Protein/Peptide	62.8/-	91.2/140.2
Ni^{2+} / Zn^{2+}	51.2/63.6	69.2/80.5
αKG or NOG	72.7 (αKG)	90.3 (NOG)
Water/SO_4^{2-}	54.8/90.0	72.5/117.3
R.m.s. deviations		
Bond lengths (Å)	0.007	0.010
Bond angles (°)	0.960	1.302

[a] Highest-resolution shell is shown in parentheses.

Supplementary Table 3. Sequences of primers used in this study

**Primers for genotyping**	
jmj13-F	5′-TCAAGTTCCATTTGCTGCTTC-3′
jmj13-R	5′-GGAATGTTTGCTGACACTTGG-3′
T-DNA specific sequence	5′-TGGTTCACGTAGTGGGCCATCG-3′
**Primers for cDNA cloning**	
*JMJ13*c-F	5′-CACCATGGCGGAAAGGAGGATCTGCTTGTC-3′
*JMJ13*c-R	5′-GTCTGAAAGTGAAAGGGCTAATAAAGCCG-3′
**Primers for mutagenesis and truncation**	
JMJ13-mu-F	5′-GCCTGGGCTGTTGCGGACCATTATTTGTAC-3′
JMJ13-mu-R	5′-GTACAAATAATGGTCCGCAACAGCCCAGGC-3′
JMJ13-ΔJmjC F	5′-AACCGAGTACCGTTGCTTCCTC-3′
JMJ13-ΔJmjC R	5′-TGGGTCACCTGGTGCAGATGA-3′
JMJ13-ΔZF- F	5′-TTTGAGAAGGAAGATGGAGTTTCAG-3′
JMJ13-ΔZF- R	5′-CAAACTGCAGACTATGGTTCCAT-3′
**Primers for genomic DNA cloning**	
*JMJ13*g-F	5′-CATGTCACAACCCATATCACAA-3′
*JMJ13*g-R	5′-GAACAACTTCCACCTACTAGAACC-3′
**Primers for real time-PCR analysis**	
JMJ13-F	5′-CAAGAGAGTCTGACCATCATCAGGAAC-3′
JMJ13-R	5′-GCATCTTCACGTCACTTTGTTGCTGAG-3′
SVP-F	5′-CTAACTGTGTGTTTACATGATCATATATAGGCA-3′
SVP-R	5′-CTTATACATGGTATCAAGTAATTTTCTTAACAACCT-3′
CO-F	5′-TGCAGCGTACCACAGACGAGC-3′
CO-R	5′-TGTATGCGTTATGGTTAATGGAAC-3′
UBC-F	5′-TCAAATGGACCGCTCTTATC-3′
UBC-R	5′-CACAGACTGAAGCGTCCAAG-3′
