# Supplementary material for: The Arabidopsis H3K27me3 demethylase JUMONJI 13 is a temperature and photoperiod dependent flowering repressor
Source: Nat Commun. 2019 Mar 21;10:1303. doi: 10.1038/s41467-019-09310-x (PMC6428840; doi:10.1038/s41467-019-09310-x)
Supplement: Supplementary file 3 — Description of Additional Supplementary Files [file 41467_2019_9310_MOESM3_ESM.pdf]

**Description of Additional Supplementary Files**

File Name: Supplementary Data 1
Description: List of differentially expressed genes in Col and jmj13.
