# Supplementary material for: The Arabidopsis H3K27me3 demethylase JUMONJI 13 is a temperature and photoperiod dependent flowering repressor
Source: Nat Commun. 2019 Mar 21;10:1303. doi: 10.1038/s41467-019-09310-x (PMC6428840; doi:10.1038/s41467-019-09310-x)
Supplement: Supplementary file 5 — Reporting Summary [file 41467_2019_9310_MOESM5_ESM.pdf]

nature research

Corresponding author(s): Daye Sun, Xiaofeng Cao, Jiamu Du

Reporting Summary

Nature Research wishes to improve the reproducibility of the work that we publish. This form provides structure for consistency and transparency in reporting. For further information on Nature Research policies, see Authors & Referees and the Editorial Policy Checklist.

Statistical parameters

When statistical analyses are reported, confirm that the following items are present in the relevant location (e.g. figure legend, table legend, main text, or Methods section).

n/a	Confirmed	
☐	☒	The exact sample size (*n*) for each experimental group/condition, given as a discrete number and unit of measurement
☐	☒	An indication of whether measurements were taken from distinct samples or whether the same sample was measured repeatedly
☐	☒	The statistical test(s) used AND whether they are one- or two-sided<br>*Only common tests should be described solely by name; describe more complex techniques in the Methods section.*
☒	☐	A description of all covariates tested
☐	☒	A description of any assumptions or corrections, such as tests of normality and adjustment for multiple comparisons
☐	☒	A full description of the statistics including central tendency (e.g. means) or other basic estimates (e.g. regression coefficient) AND variation (e.g. standard deviation) or associated estimates of uncertainty (e.g. confidence intervals)
☐	☒	For null hypothesis testing, the test statistic (e.g. *F*, *t*, *r*) with confidence intervals, effect sizes, degrees of freedom and *P* value noted<br>*Give P values as exact values whenever suitable.*
☒	☐	For Bayesian analysis, information on the choice of priors and Markov chain Monte Carlo settings
☒	☐	For hierarchical and complex designs, identification of the appropriate level for tests and full reporting of outcomes
☒	☐	Estimates of effect sizes (e.g. Cohen's *d*, Pearson's *r*), indicating how they were calculated
☐	☒	Clearly defined error bars<br>*State explicitly what error bars represent (e.g. SD, SE, CI)*

Our web collection on statistics for biologists may be useful.

Software and code

Policy information about availability of computer code

Data collection	HKL2000/3000, X-ray data collection and processing;
Data analysis	Coot, model building; Phenix, structure refinement; Molprobity, structure geometry analysis; SOLOMON, density modification; Data Explorer, mass spectrum data analysis; fastQC, sickle, Tophat2, htseq-count, DEGseq, RNA seq analysis; SAMtools, MACS, Bowtie 2, ChIP-seq analysis.

For manuscripts utilizing custom algorithms or software that are central to the research but not yet described in published literature, software must be made available to editors/reviewers upon request. We strongly encourage code deposition in a community repository (e.g. GitHub). See the Nature Research guidelines for submitting code & software for further information.

Data

Policy information about availability of data

All manuscripts must include a data availability statement. This statement should provide the following information, where applicable:

- Accession codes, unique identifiers, or web links for publicly available datasets
- A list of figures that have associated raw data
- A description of any restrictions on data availability

X-ray structures have been deposited in the RCSB Protein Data Bank with the accession codes: 6IP0 [https://www.rcsb.org/structure/6IP0] for the JMJ13-alpha-KG

complex and 6IP4 [https://www.rcsb.org/structure/6IP4] for the JMJ13–NOG–H3K27me3 complex. RNA-Seq and ChIP-Seq data have been uploaded to NCBI SRA with accession number SRP168443 [https://www.ncbi.nlm.nih.gov/sra/SRP168443] and SRP174856 [https://www.ncbi.nlm.nih.gov/sra/ SRP174856], respectively. The source data underlying Fig. 1, Fig. 3, Fig. 5, Supplementary Fig. 1, and Supplementary Fig. 5-10 are provided as a Source Data file.

Field-specific reporting

Please select the best fit for your research. If you are not sure, read the appropriate sections before making your selection.

☒ Life sciences ☐ Behavioural & social sciences ☐ Ecological, evolutionary & environmental sciences

For a reference copy of the document with all sections, see nature.com/authors/policies/ReportingSummary-flat.pdf

Life sciences study design

All studies must disclose on these points even when the disclosure is negative.

Sample size	In vivo demethylase activity analysis more than 30 pairs of transfected nuclei versus non-transfected nuclei in the same field of view were observed and quantifications statistical analyzed. For phenotyping of leaf numbers, we used at least 15 healthily grown plants grown under controlled conditions for data collection. For realtime PCR experiments, we collected ten days seedlings from 30 plants as one sample to isolate the total RNA, and three independent collections were performed for three independent biological repeats, respectively.
Data exclusions	No data were excluded.
Replication	For Mass Spectrum-based in vitro activity assay, the experiments were repeated 3 times. For the flowering time statistics, the real-time PCR and the western blotting experiments were repeated 3 times.
Randomization	For flowering phenotype, the plants were grown in a completely randomized manner in the growth chamber. The samples were allocated randomly.
Blinding	No blinding was used.

Reporting for specific materials, systems and methods

Materials & experimental systems

n/a	Involved in the study	
☒	☐	Unique biological materials
☐	☒	Antibodies
☒	☐	Eukaryotic cell lines
☒	☐	Palaeontology
☒	☐	Animals and other organisms
☒	☐	Human research participants

Methods

n/a	Involved in the study	
☐	☒	ChIP-seq
☒	☐	Flow cytometry
☒	☐	MRI-based neuroimaging

Antibodies

Antibodies used	Antibodies used in this study are: H3K27me3: Millipore 07-449; H3K27me2: Millipore 07-452; H3K27me1: Millipore 07-448; H3K4me3: Millipore 07-473; H3K4me2: Millipore 07-030; H3K4me1: Millipore 07-436; H3K9me3: Millipore 07-442; H3K9me2: Millipore 07-441; H3K9me1: Millipore 07-450, 1:100; H3K36me3: Abcam ab9050; H3K36me2: Millipore 07-274; H3K36me1: Millipore 07-548; H3 Abcam ab1791.
Validation	Antibodies used in this study are commercially available,the specificity had been tested by the supplier.

ChIP-seq

Data deposition

☒ Confirm that both raw and final processed data have been deposited in a public database such as GEO.

☐ Confirm that you have deposited or provided access to graph files (e.g. BED files) for the called peaks.

Data access links
May remain private before publication.

https://www.ncbi.nlm.nih.gov/sra/?term=SRP174856

Files in database submission

1Ab: 22℃ SD Wild type ChIP-seq anti-H3K27me3; 2Ab: 22℃ SD mutant of jmj13 ChIP-seq anti-H3K27me3;3Ab: 22℃ LD Wild type ChIP-seq anti-H3K27me3;4Ab: 22℃ LD mutant of jmj13 ChIP-seq anti-H3K27me3;5Ab: 28℃ SD Wild type ChIP-seq anti-H3K27me3;6Ab: 28℃ SD mutant of jmj13 ChIP-seq anti-H3K27me3.

Genome browser session
(e.g. UCSC)

NA

Methodology

Replicates

NA

Sequencing depth

ChIP-seq were all sequenced in single end. Specifically, ChIP-seq of H3K27me3.Col.22LD were sequenced with 11,685,444 raw reads and 10,105,412 of these uniquely mapped to the genome; H3K27me3.Col.22SD with 13,093,273 raw reads and 11,366,478 uniquely mapped; H3K27me3.Col.28SD with 17,921,411 raw reads and 15,036,589 uniquely mapped; H3K27me3.jmj13.22LD with 15,663,333 raw reads and 13,670,693 uniquely mapped; H3K27me3.jmj13.22SD with 14,013,209 and 12,108,982 uniquely mapped; H3K27me3.jmj13.28SD with 14,157,086 raw reads and 12,225,878 uniquely mapped.

Antibodies

anti- H3K27me3 (Millipore 07-449), anti-H3 (Abcam; ab1791)

Peak calling parameters

macs2 callpeak --nolambda --fix-bimodal -q 0.001 --extsize 200 -g dm -t chips.bam

Data quality

Qualified peaks were filtered by GNU gawk with peak score >= 50 (p value <= 1e-5).

Software

Duplicated reads and low-mapping quality reads were identified and removed with SAMtools. Enriched intervals were identified by MACS version 2.1.0 with default parameters.
